# SPION Conjugated Curcumin Nano-Imaging Probe: Synthesis and Bio-Physical Evaluation

**Published:** 2019

**Authors:** Rasoul Rahimnia, Zeinab Salehi, Mehdi Shafiee Ardestani, Hamid Doosthoseini

**Affiliations:** a *School of Chemical engineering, College of Engineering, University of Tehran, Tehran, Iran. *; b *Department of Medicinal Chemistry, Faculty of Pharmacy, University of Tehran, Tehran, Iran.*

**Keywords:** Magnetic nanoparticles, Curcumin, MRI, Citric acid, Functionalization MNPs

## Abstract

In this work, we investigated the loading and conjugation of Curcumin on oleic acid (OA) and citric acid (CA) functionalized iron oxide nanoparticles and its applications in improving contrast in MRI. Magnetic iron oxide nanoparticles (Fe_3_O_4_, MNPs) were synthesized using the co-precipitation method and characterized by XRD, DLS, FT-IR, VSM, and SEM. FT-IR results confirmed functionalization with oleic acid and citric acid. Curcumin was loaded and conjugated with the Nano-Systems and the amount of Curcumin loaded was quantified using spectrophotometry at 419 nm wavelength. The impact of solvent on the loading of Curcumin was studied. The wt% of loaded Curcumin was found to be 0.189 wt% using dimethylformamide (DMF) whereas using a combination of water-ethanol (15% v/v), this increased to 56.149 wt%. T2 relaxation time was determined using a 1.5 Tesla MRI machine; results showed that the MNPs reduced T2. Cytotoxicity of Nano-Systems (NS) in MTT assay showed that concentrations higher than 80 μg/mL (C_NS_ > 80 μg/mL) could lead to cancer cell death and low concentrations, up to 40 μg/mL (C_NS_ < 40 μg/mL) could be evaluated for diagnostic purposes.

## Introduction

Magnetite nanoparticles (MNPs) are a large class of nanomaterials that have received increased attention during the last decade due to their unique properties. Most significantly, the ability to induce magnetic moments in the presence of an external magnetic field make them a potent option for therapeutic and diagnostic uses in the biomedical field (1-5). The applicability of MNPs ranges from contrast agents in magnetic resonance imaging (MRI) and magnetic enhanced enzyme-linked immunoassays to drug delivery agents and hyperthermia applications. Remote controlling and the ability of tracking the MNPs from outside of the body is considered a significant advantage (5, 6). 

MNPs with high magnetization value, small size, and special surface coating are suitable for these purposes (7-9).

Magnetic resonance imaging (MRI) is a common tool for diagnosis of tumors and cancer. Nuclear magnetic resonance phenomenon is the basis for MRI with Hydrogen as one of the most appropriate elements for nuclear magnetic resonance effect. This atom found in abundance in biological systems including the human body and, thus, MRI can provide clear images of anatomical detail of both bones and soft tissues. MRI often produces more legible images than other methods. The ability to scan without radiation is an important advantage of this technique. Using contrast agents in MRI is paramount for detecting tumors and cancer. Exploring possible new contrast agents for MRI is one of the most pursued and important topics of research. MNPs, due to their unique properties such as their nanoscale structure and magnetism, were developed as contrast agents for MRI (10). These particles act like magnets only in the presence of an external magnetic field and do not hold any residual magnetism upon elimination of the external magnetic field (5). As such, they are able to significantly enhance the sensitivity of diagnosis using MRI (10). MNPs are not stable at normal physiological conditions and show an inherent quality to aggregate due to their hydrophobic nature; they must therefore undergo surface modification with suitable biologically active specific functional groups prior to use (5). The first patented use of MNPs as a contrast agent was registered in 1998 by Hesegawa *et al*. for dextran coated MNPs (11).

Magnetic nanoparticles have been shown to be applicable as cancer treatment in three different ways: conjugation of specific antibodies to selectively bind to tumor receptors and inhibit growth, hyperthermia for tumor therapy using targeted MNPs, and loading of anticancer drugs onto the MNPs (12, 13).

Using MNPs as carriers provides both a physical and chemical targeting method for drug delivery. By using an external magnetic field drugs loaded or conjugated to MNPs could be delivered to the specific location easily (5, 14), functionalized MNPs could selectively attach to specific cells to locally deliver loaded or conjugated drug (15) and the surface-bound drugs can be released from MNPs by changing the physiological conditions, and taken up by the affected cells (5). In order to achieve loading or conjugation of a specific antibody or drug to the MNPs, a functionalization process is necessary (16). Functionalization and surface coatings of MNPs provide a steric barrier preventing them from agglomeration and opsonization (the uptake by the reticuloendothelial system (RES), thus shortening circulation time in the blood and enabling MNPs to target the drug to specific sites reducing side effects). In addition, coatings and functionalization provide a means to modify the surface properties of MNPs such as surface charge and chemical activity (7).

Citric acid is a small molecule and has been shown to be suitable for facile functionalization; the use of citric acid also overcomes the steric inhibition that large molecule surfactants and long polymer chains exhibit for drug loading (17). Citric acid gives hydrophilic properties to magnetic nanoparticles (18), having three carboxyl functional groups, one or two carboxyl groups absorb on the surface of the nanoparticles and at least one remains free (19). Nanoparticles coated with citric acid have been commercially produced since 2002 by co-precipitation method using excess citric acid to allow maximum loading, with the commercial name VSOP C184 was and have been used in clinical studies for diagnostic characterization (20).

**Table 1:** Results of loaded Curcumin on MNPs that functionalized with different method

**Sample name**	**MNP**	**Solvent**	**Activator**	**µg Cur/g Nano system**
CUR1	MNP-OA	DMF	DCC	0
CUR2	MNP-CA2	DMF	DCC	0. 63 ± 0.11
CUR3	MNP-CA1	DMF	DCC	1.89 ± 0.38

**Table 2:** Results of loaded Curcumin on functionalized MNP by changing in Curcumin solvent

**Sample name**	**MNP**	**Solvent**	**Activator**	**µg Cur/g Nano system**
CUR4	MNP-CA1	DMSO	DCC	2.67± 0.133
CUR5	MNP-CA1	DMSO	EDC/NHS	2.39± 0.129
CUR6	MNP-CA1	Ethanol	EDC/NHS	5.46± 1.247
CUR7	MNP-CA1	Water (pH=12-13)	EDC/NHS	10.57± 2.126
CUR8	MNP-CA1	Water/Ethanol (30% v/v)	EDC/NHS	167.45± 3.312

**Table 3:** DLS results of MNP-COOH

**Sample**	**Hydrodynamic diameter (nm)**	**PDI**	**Zeta Potential (mV)**
MNP-COOH	37.7 ± 6.52	0.558	-15.4 ± 5.01

**Table 4:** T2 signal value obtain from curve fitting in Figure 12

**C** **NS ** **(µg/mL)**	**225**	**175**	**100**	**50**	**20**	**Pure Water**
1/T2 (ms-1)	0.021±0.0008	0.016±0.0019	0.01±0.0011	0.006±0.0006	0.004±0.0009	0.0004±0.0001
T2 (ms)	47.62±1.81	62.5±7.42	100±11	166.67±16.67	250±56.25	2500±625

**Figure 1:**
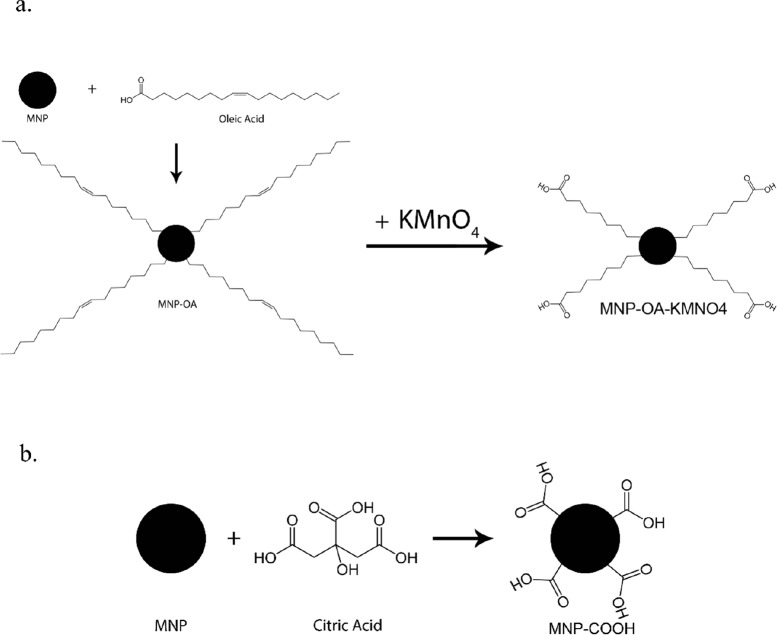
Mechanism of MNPs functionalization with a. Oleic Acid ,b. Citric Acid

**Figure 2:**
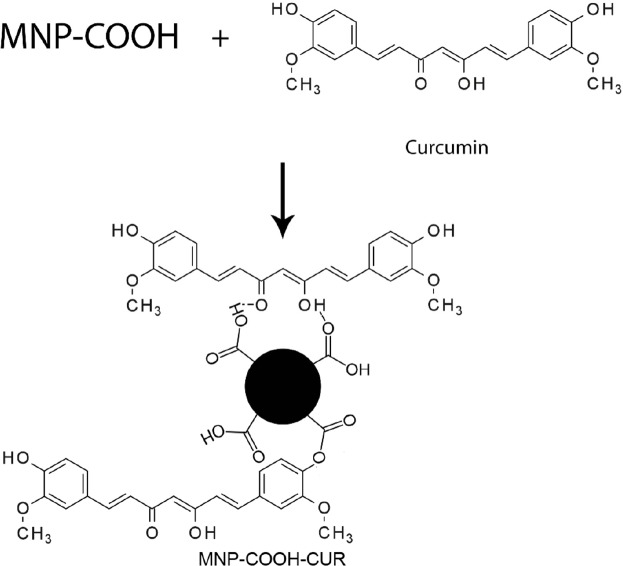
Interaction between curcumin and functionalized MNPs can be hydrogen bond or conjugation of functional groups

**Figure 3:**
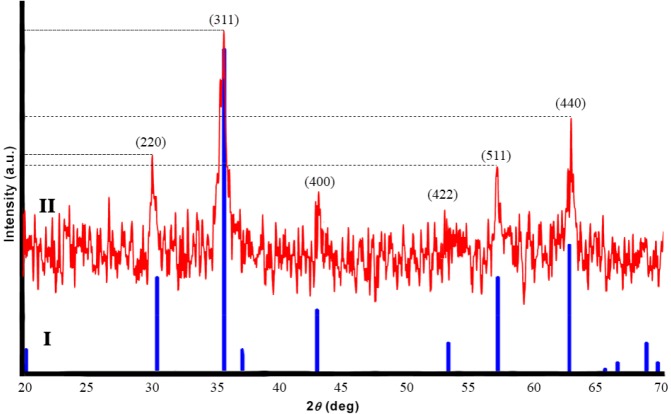
Physical characterization of magnetic nanoparticle formulations with XRD: pure Fe3O4 (I, Blue) compared with synthesized Fe3O4 (II, Red)

**Figure 4:**
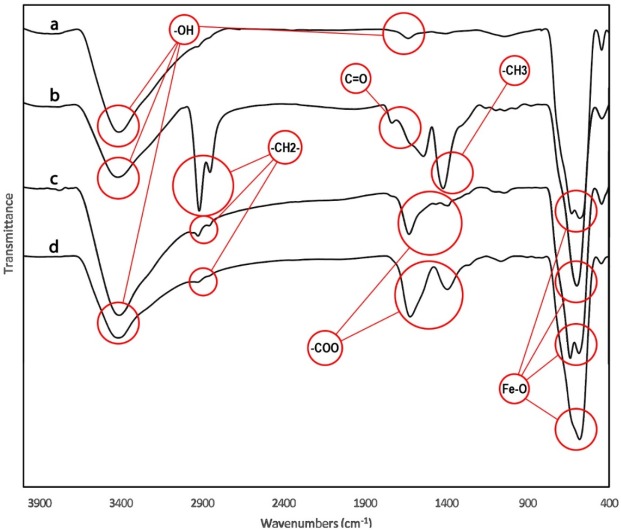
Fourier transform infrared spectra of a) MNP, b) MNP-OA, c) MNP-OA-KMNO4 and d) MNP-COOH. Data was obtained for dry powder samples. Characteristic adsorption bonds are marked on the Figure

**Figure 5:**
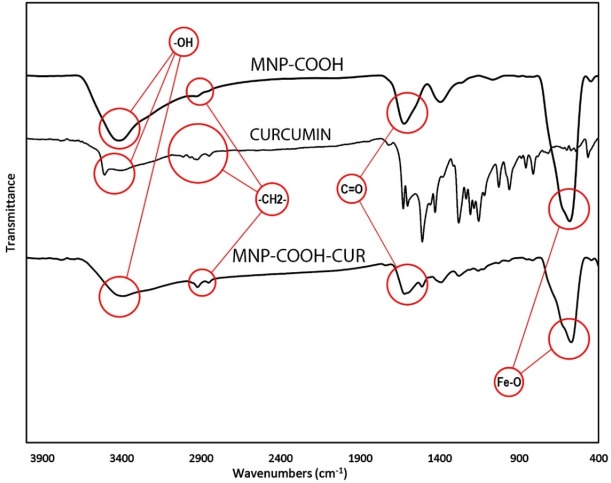
Characterization of MNP-COOH, Curcumin and MNP-COOH-CUR with FTIR spectra. Characteristic adsorption bonds are marked on the Figure

**Figure 6:**
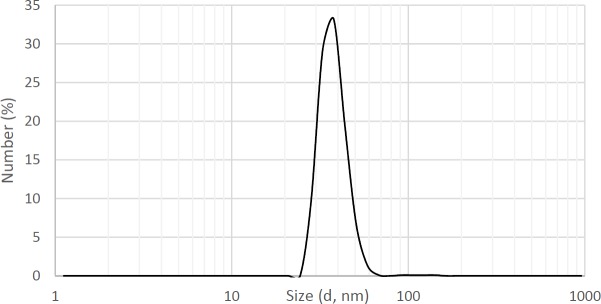
Dynamic light scattering measurement of MNPs-COOH. Data demonstrates the average hydrodynamic size is 37 nm. Data represents the average of three replicates

**Figure 7:**
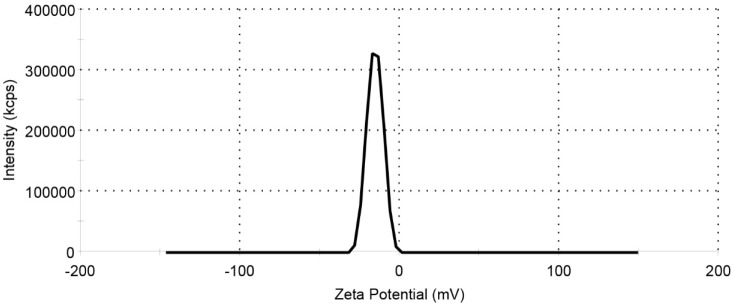
Zeta potential of MNPs-COOH

**Figure 8:**
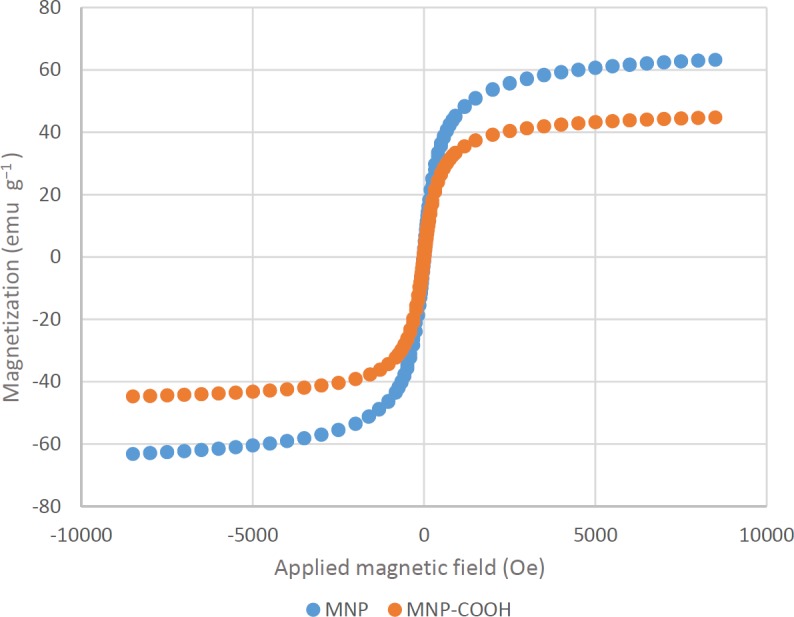
Magnetization of MNPs and MNP-COOH as a function of the applied magnetic field

**Figure 9:**
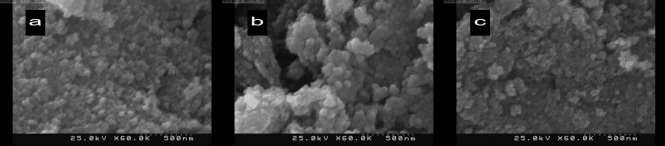
SEM image of MNPs (a), MNPs-COOH (b) and MNPs-COOH-Cur (c)

**Figure 10:**
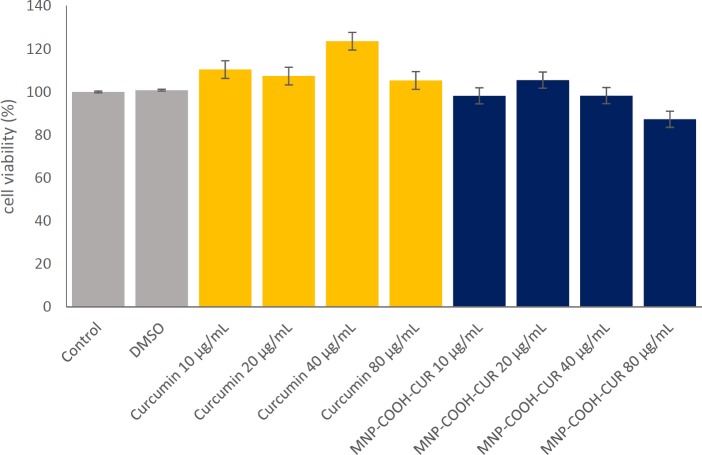
Cell viability data of MDA-MB-231 cells after incubation 24 h with different curcumin and MNP-COOH-CUR concentration. The error bars show standard deviation. The significance level for the statistical analysis was 0.05. Curcumin in different concentration causes an insignificant increase in cell growth (*p* > 0.05). We have seen toxicity and a significant decrease in the number of cells for MNP-COOH-CUR in 80 µg/mL concentration (*p* < 0.05). Comparisons made with the control group of untreated cells. There was no significant difference between the other groups with the control group

**Figure 11:**
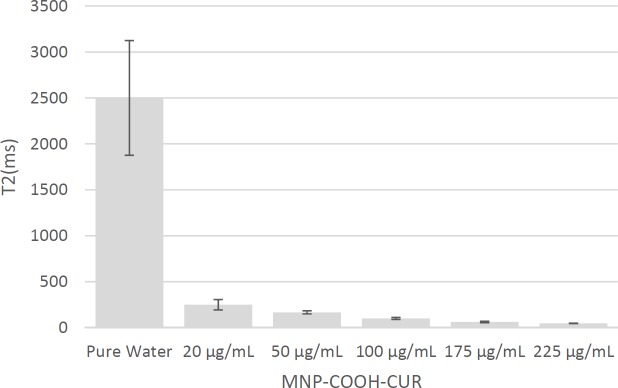
T2 signal intensity of samples with different MNP-COOH-CUR concentration. The error bars show standard deviation

**Figure 12:**
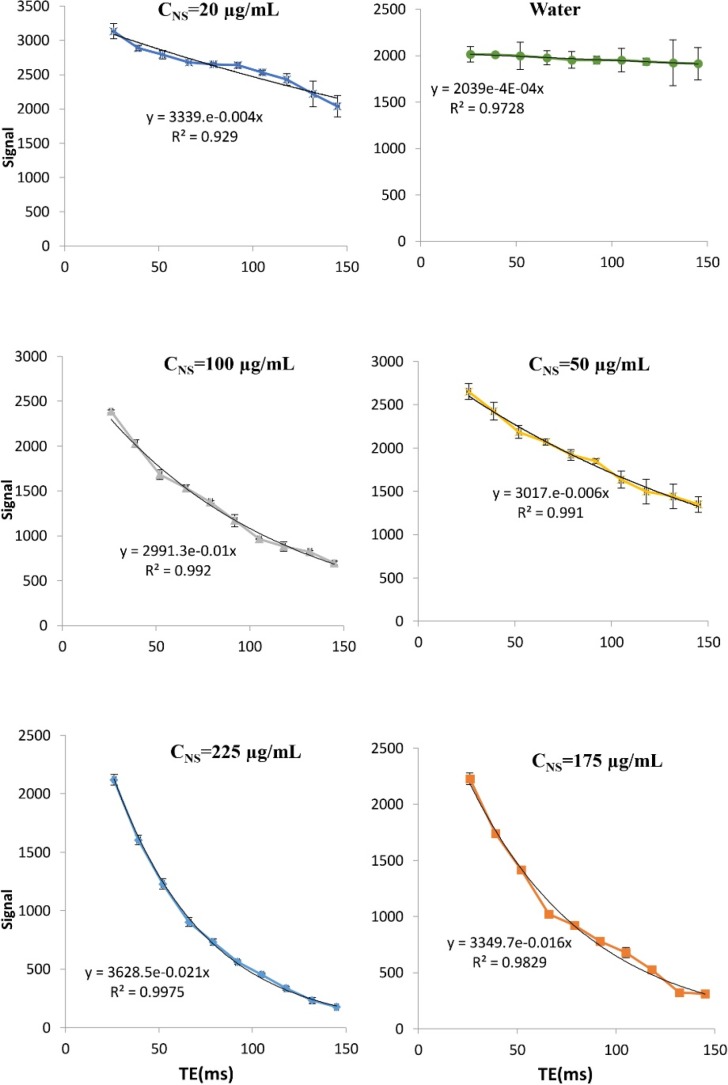
Magnetic resonance image (MRI) characteristics of magnetic nanoparticle. Signal intensities for pure water and different nano-system (MNP-COOH-CUR) concentration as a function of echo delay time. The error bars show standard deviation

In the last few decades, herbal medicines and the chemicals they contain have received significant attention for their potential therapeutic properties, including for cancer treatment. One such herbal medicinal ingredient is turmeric. Turmeric is a bright yellow aromatic powder obtained from the rhizome of *Curcuma longa (L)*, used for flavoring and coloring in Asian cooking and formerly as a fabric dye. One of the most important class of chemical substances found in turmeric are Curcuminoids, this group includes Curcumin (diferuloylmethane), demethoxy Curcumin, and bis demethoxy Curcumin. Curcumin is the compound that has shown most therapeutic potential and has received the most attention for study and research; it constitutes 3-4% of powdered turmeric. Curcumin itself appears as crystalline compound with a bright orange-yellow color (21, 22). The therapeutic properties of Curcumin experimentally investigated showing its anticancer, anti-tumor, anti-inflammatory and anti-oxidant properties (21, 23-26) among others and rendering it an interesting compound for medical applications. (27).

Conjugation or loading of Curcumin onto different platforms in order to boost its therapeutic effects is a topic of high interest. Studies conducted in recent years have paid significant attention to using polymers for Curcumin loading (26). In 2010 Rocktotpal Konwarh *et al*. used polyethylene glycol (28) and Lam Dai Tran *et al.* used chitosan (7) for nanosized magnetofluorescent Fe_3_O_4_ coating and loaded Curcumin by allowing diffusion into this layer, Fengxia Li *et al*. used poly lactic acid for Curcumin loading in 2011 (29). Another study in the same year, presented methods to create magnetic fluids created by coating nanoparticles with dextran and pluronic polymer; these were further loaded with Curcumin for sustained release Curcumin and contrast agent in MRI (30). Ning Wang *et al*. in their study used PEO-PPO-PEO copolymers for nanoparticles coating and Curcumin entrapped in coated nanoparticles structures (31). Kwok Kin Cheng *et al*. entrapped Curcumin in the structure of the magnetic nanoparticles coated with polyethylene glycol and poly-lactic (32). Also Mancarella *et al*. used entrapment of Curcumin in magnetic nanoparticles coated with dextran and poly-lysine(33).

In this study, the surface of synthesized Fe_3_O_4_ nanoparticles was modified using oleic acid (OA) and citric acid (CA) to conjugate Curcumin without the use of any polymer coating, thus allowing for smaller sized nanoparticles with hypothesized higher efficacy. Functionalized MNPs compared and evaluated to increase Curcumin loading. These nano-bio systems further characterized and analyzed *in-vitro* for MRI and MTT assay for cytotoxicity on cancer cells. The purpose of this study is to enhance the loading of curcumin on MNPs by improved method to present a novel smaller and more magnetically active nanoparticle for diagnostic purposes and eventually this nanosystem be further studied as a potential cancer treatment. The current study integrates body science, chemical engineering, pharmacy, and nanotechnology.

## Experimental

*Materials*

Iron (II) sulfate heptahydrate (FeSO_4_.7H_2_O, 99%), iron (III) chloride hexahydrate (FeCl_3_.6H_2_O, 99%), sodium hydroxide (98%, anhydrous pellets), Curcumin (purity > 99%), dimethylformamide (DMF) (99%), acetone, methanol 96%, oleic acid, Potassium permanganate (KMnO_4_), Dimethyl sulfoxide (DMSO), N,N›-Dicyclohexylcarbodiimide (DCC, and citric acid (CA) were purchased from Merck and ethyl (dimethylaminopropyl) carbodiimide/N-hydroxysuccinimide (EDC/NHS) was purchased from Sigma-Aldrich. Deionized water was used throughout all experiments. 

*Synthesis of functionalized magnetic nanoparticles*

*Functionalized magnetic nanoparticles with oleic acid (MNP-OA)*

To remove dissolved oxygen, 100 mL of deionized water was degassed for 30 min using nitrogen gas (34). Fe^2+^ and Fe^3+^ ions were added with a ratio 2: 1 (0.78 g FeSO_4_.7H_2_O and 1.44 g FeCl_3_.6H_2_O) on a magnetic stirrer at 500 rpm. The temperature gradually increased to 80 °C to ensure complete dissolution of salts. Subsequently, the 1 M NaOH solution added to the mixture of iron salts and stirring rate raised to 1000 rpm. At pH about 12 the solution changed color to black and the reaction was maintained at these conditions for another 30 min to ensure reaction completion (17, 35). Subsequently, 0.6 g Oleic acid was added drop by drop to MNP solution and the reaction continued for one hour at 70 °C. The nanoparticles were then separated using a stationary magnet and washed 3 times with deionized water and a also 3 times with ethanol. Oleic acid on the surface of MNPs oxidized with KMnO_4_ to establish a carboxyl group on the surface of MNPs. For this purpose, 20 mL of KMnO_4_ solution with a concentration of 10 mg/mL was added to the oleic acid coated nanoparticles. The reaction was carried out for 24 h at room temperature; MNPs were subsequently separated with a magnet and washed 3 times with deionized water (36). The surface modifications in each step (MNP-OA, MNP-OA-KMNO4 and MNP-COOH) were investigated with FT-IR analysis. The mechanism of functionalization has been seen in Figure 1 (a).

*Functionalized magnetic nanoparticles with citric acid*

*One pot (MNP-CA1)*

Fe_3_O_4_ nanoparticles were prepared by a co-precipitation method. In a typical synthesis, 1.08 g of FeCl_3_.6H_2_O and 0.565 g of FeSO_4_.7H_2_O were dissolved in 80 mL of degassed deionized water in a round bottom flask and the temperature gradually increased to 70 °C under nitrogen purging with constant stirring at 1000 rpm. The temperature maintained at 70 °C for 30 min after which 20 mL of ammonia solution added instantaneously to the reaction mixture. The pH increased to 12 and the reaction chamber was kept at the same temperature for another 30 min. Then 3.5 g citric acid was added to the above reaction mixture and reaction temperature gradually increased to 90 °C and kept at 90 °C for 60 min with continuous stirring. The reaction mixture then cooled to room temperature and the black colored precipitates were separated and thoroughly rinsed with water. During each rinsing step, the samples were separated from the supernatant using a permanent magnet (17, 37). Citric acid was anchored on the surface of freshly prepared MNPs by the direct addition method as shown in Figure 1(b).

*Two pot (MNP-CA2)*

In this method before the addition of citric acid, MNPs separated using a magnet and washed with deionized water. Subsequently, MNPs were dispersed in another aqueous solution with pH adjusted to about 3 using HCl, 3.5 g citric acid was added. The temperature gradually raised to 90 °C and then kept at 90 °C for 60 min with continuous stirring. After this time, MNP-COOH were separated with a magnet and washed with water (38). The mechanism is similar to the previous method as shown in Figure 1(b).

*Preparation of Curcumin conjugated to magnetic nanoparticles*

To investigate the effect of solvent and activator on the Curcumin loading, DMF, DMSO, Ethanol, Alkaline Water (pH = 12-13) and Water/Ethanol (30% v/v) as solvent and DCC and EDC/NHS as activators were tested. For these experiments, carboxylated MNPs dispersed in solvent and activator to activate carboxyl groups on the MNPs were added to the solution and stirred for 1 h. After that, 0.02 g Curcumin was added and, to prevent degradation of Curcumin by light, the flask was covered with aluminum foil. The reaction solution was kept in these conditions, under constant stirring for 1 day. After this time, samples separated with a magnet and rinsed with acetone to wash physically adsorbed Curcumin. In this process, conjugated Curcumin remained on samples and washed. The samples then was dried at 50 °C for 4 h. The mechanism is shown in Figure 2.

*Curcumin loading efficiency*

In order to measure the Curcumin loading efficiency, a certain amount of the nano system was dispersed in acetone for 3 h, MNPs then was separated with a magnet. The amount of Curcumin in solution was measured by spectrophotometry of Unit Company at 429 nm wavelength. 

*Characterization of nanoparticles*

The phase structure of MNPs was studied by x-ray diffraction. Infrared (IR) spectra was recorded with a VECTOR 2000 FTIR Spectrometer, using KBr pellets, in the range of 400–4000 cm^−1^. Scanning Electron Microscope (SEM) of images was used to determine the morphology and particle size of MNPs. The magnetizations versus field was measured with a vibrating sample magnetometer (VSM) and evaluated in terms of saturation magnetization and coercivity. The hydrodynamic size of MNPs in water was determined by dynamic light scattering (DLS).

*Cell viability test of nanoparticles*

The cytotoxicity of the MNP-COOH-CUR was evaluated by determining the viability of MDA-MB-231 after incubation in medium containing the MNPs at concentrations 10, 20, 40, and 80 µg/mL. The control experiments were carried out using the complete growth culture media without MNPs. The cell viability testing was carried out via the reduction of the MTT reagent (3-[4,5-dimethyl-thiazol-2-yl]-2,5-diphenyltetrazolium bromide). The MTT assay was performed in a 96-well plate following the standard procedure (39) with minor modifications. The cells seeded at a density of 6000 cells per well and incubated at 37 °C for 24 h in the medium containing the nanoparticles. The culture medium in each well was then removed and after that, 200 µL of medium with concentration 0.5 mg/mL of MTT was added to each well. After 4 h of incubation at 37 °C, the medium was removed and the formazan crystals were dissolved with DMSO for 15 min. The optical absorbance was then measured at 540 nm on a microplate reader.

Generally, nanoparticles adsorption in cells is fast and in case of cancer cells adsorption is even faster. For example, this time for iron nano particles is less than 6 h and for components such as MNPs this time is even shorter, so cytotoxicity will be shown in less than 12 h or at most in 24 h. Therefore, there is no need to check cytotoxicity after 24 h unless the cytotoxicity isn’t shown.

*In-vitro MRI test*

MRI experiments were performed in a clinical magnetic resonance (MR) scanner (1.5 T). MNP-COOH-CUR were suspended in tubes of water at concentration of 20, 50, 100, 175, 225 mg/mL. The tubes placed into the MR scanner and a number of MR sequences were run. T2 relaxation times were determined from a multi-echo spin-echo (SE) sequence (10 echoes; repetition time (TR): 3000 ms). The relaxation rates for each sample were computed using Dicomwork (V 1.3.05), Microdicom (V 0.7.1.1824), MATLAB (V 1.0.0.1), and Microsoft Office Excel 2007.

## Results and Discussions

*MNPs Characterization*

*XRD Analysis*

Figure 3 shows XRD patterns of pure Fe_3_O_4_ (I, Blue) and synthesied Fe_3_O_4_ (II, Red). Six characteristic peaks for Fe_3_O_4_ corresponding to (220), (311), (400), (422), (511), and (440) were observed in the sample (40). These peaks reveal that the resulting nanoparticles were pure Fe_3_O_4_.

*FT-IR Analysis of functionalized MNPs*

The MNPs was further characterized by FTIR to confirm the chemical binding. FT-IR spectra of MNPs shown in Figure 4a cursory inspection of the spectra shows two absorption bands below 1000 cm^-1^ as a common feature of all the ferrites. Absorption in this region is not restricted to this class of compounds but occurs in the spectra of most metal oxides (41). In all spectra the band at 580 cm^-1^ corresponds to the vibration of the Fe–O bonds in the crystalline lattice of Fe_3_O_4_ (42). During preparation of Fe_3_O_4_ nanoparticles by the chemical co-precipitation, their surfaces were readily covered with hydroxyl groups in an aqueous environment, the characteristic bands of hydroxyl groups, 1630 and 3405 cm^-1^, appear in the FTIR spectrum Figure 4a (43, 44). The bands at 2852 and 2922 cm^-1^ are attributed to the asymmetric CH_2_ stretch and the symmetric CH_2_ stretch in oleic acid, respectively, and the band at 1409 cm^-1^ corresponds to the CH_3_ umbrella mode of oleic acid and the band at 1710 cm^-1^, corresponds to stretching vibration of C = O in oleic acid (Figure 4b). After using KMnO_4_ on MNP functionalized with oleic acid the product nanoparticles (MNP-OA-KMNO4) were analyzed with FTIR. Two bands at 1457 and 1523 cm^-1^ appeared as seen in Figure 4c and were attributed to the asymmetric (–COO) and symmetric (–COO) stretch vibration band, that confirmed the carboxyl group on it. FTIR of MNP-COOH shown in Figure 4d that two bands at 1457 and 1523 cm^-1^ confirmed carboxylation of MNPs.

*Curcumin Loading*

To investigate the effect of solvent and activator on Curcumin loading, the Curcumin on the functionalized MNPs was released through treatment with acetone, quantified, and compared. Loading obtained in DMF (CUR1, CUR2 and CUR3) was very low, the produced nanoparticles analyzed by FT-IR and almost no change was observed. The amount of loaded Curcumin less than the resolution of the device could be the reason for this observation. The release data of these samples (CUR1, CUR2 and CUR3) were collected in Table 1. According to these results, the most Curcumin loading was related to MNP-CA1, so this sample was selected for further analyses. In the following of the text MNP-COOH representes MNP-CA1.

In order to increasing the Curcumin loading, DMSO, ethanol, water, ethanol/water as the solvent and DCC, EDC/NHS as activator were tested. The results of these tests are shown in Table 2 (CUR4, CUR5, CUR6, CUR7 and CUR8) and illustrate an increased amount of Curcumin loaded on the MNPs using a mixture of water and ethanol as solvent (CUR8). This sample collected for further analyzed with FT-IR. 

*FT-IR Analysis of MNP-COOH-CUR*

Figure 5 shows the FTIR spectra of MNPs-COOH, Curcumin and MNPs-COOH-CUR. The absorption peaks around 563 or 580 cm^-1^ in Figure 5 are the characteristic absorption of Fe-O bond of magnetite nanoparticles (41). The absorption bands at 2920-2850 cm^-1^ ascribed to the C-H stretching vibrations, which is indicative of citric acid and Curcumin on MNPs. A large and intense band around 3400 cm^-1^ is assigned to the structural OH groups on the citric acid, Curcumin and MNPs (45). The 1600 cm^-1^ peak is assignable to the C = O vibration (symmetric stretching) from the COOH group of citric acid and indicate ester linkage between citric acid and Curcumin.

*DLS Analysis*

As MNPs are widely used in medical and diagnostic processes, the properties of these particles in the fluid environment are important and valuable to study. Hydrodynamic and rheological properties of MNPs dramatically depend on morphology and surface charge. The study of these parameters performed using DLS analysis.

As seen in Figure 6, the average hydrodynamic diameter of particles is about 37 nm. Particles with diameters between 7 to 3500 nm have been used commercially in medical science (1).

The zeta potential result of the MNPs-COOH shown in Figure 7 and Table 3. Due to the presence of carboxyl group on MNP, the value of the zeta potential is negative.

*Magnetic properties of MNPs*

The magnetic properties of MNPs were characterized by vibrating sample magnetometer (VSM). It is important to characterize the magnetic properties of MNPs before their use as a contrast agent for MRI. Figure 8 shows that the VSM curves of both MNPs and MNP-COOH rapidly approach a saturation magnetization. The absence of hysteresis in their magnetic profiles suggest that these nanoparticles are superparamagnetic. The saturation magnetization (Ms) of Fe3O4 particles` is in the range of 60–80 emu g^−1 ^and is lower than that of bulk Fe3O4 (about 90 emu g^−1^). This discrepancy in Ms can be due to the difference in particle size, as it has been reported in the literature that saturation magnetization of Fe3O4 nanoparticles decreases with reducing particle size (46). The significant decrease in Ms value of MNP-COOH is attributed to the existence of a magnetically inactive surface layer and some diamagnetic contribution from the citric acid (47).

*. SEM Analysis*

The agglomerate sizes of the MNPs are determined by a scanning electron microscope (SEM) (KYKY EM - 3200) shown in Figure 9. The average size of the agglomerated MNPs (a), MNPs-COOH (b), and MNPs-COOH-CUR (c) were 55, 71, and 77 nm, respectively. The increasing trend of agglomerate sizes of samples from 55 to 77 nm asserts the conjugation of Curcumin to functionalized MNPs.

*Cell viability test of nanoparticles*

The anticancer cytotoxic activities of MNPs were evaluated by cell viability. It was previously reported that Iron Oxide nanoparticles have no significant cytotoxicity up to 100 μg/mL (48, 49). The cytotoxicity assay carried out with MDA-MB-231 cells at different concentrations of Curcumin in solution or Curcumin was loaded in MNPs for a day by MTT assay and the results are shown in Figure 10. For the Curcumin in concentrations below 80 μg/mL the cell viability increased showing the cell protective property of Curcumin, which has been confirmed in literature (50). It found that there is no significant difference in cell viability of the MNP-COOH-CUR in the cells treated with a concentration of 40 μg/mL compared to the cells treated without nanoparticles. At a concentration of 80 μg/mL MNP-COOH-CUR, the cell viability decreased and the anticancer ability was shown. However, the comparative cytotoxicity activity between Curcumin and MNP-COOH-CUR demonstrates better therapeutic efficacy of MNP-COOH-CUR than that of Curcumin, validating the effectiveness of MNP-COOH-CUR. The authors propose that the MNP-COOH-CUR will demonstrate its anticancer properties by delivering CUR more efficiently to the cancer cells. The control cancer cells were not affected at ultrastructural level and did not show any changes in cellular integrity.

*In-vitro MRI test*

Engineered MNPs have the potential for simultaneous diagnosis and therapy in one formulation, unlike traditional contrast agents or drugs (30). We have evaluated our iron oxide formulations (MNP-COOH-CUR) for *in-vitro* MRI agent characteristics as a contrast agent and shown the effects of MNP on the relaxation time of environment. The traverse relaxation signal intensities (T2) of the pure water and water with concentration of 20, 50, 100, 175, and 225 mg/mL of MNPs versus TE (echo time) are shown in Figure 12. Signal intensity is dependent on echo time (TE) and formulated by the following equation.

Using this function and regression with respect to the data, T2 values obtained for different concentrations, are shown in Table 4 and Figure 11. These values show that the presence of MNPs led to a sharp reduction in the time T2 and an increased concentration of MNPs exaggerateds this effect.

## Conclusion

In summary, superparamagnetic iron oxide (magnetite) nanoparticles were synthesized by co-precipitation of iron ions and subsequently coated with carboxylic functional groups. To further increase the stability and biocompatibility of nanoparticles, the surface of MNPs were modified with oleic acid and citric acid (with one and two-step methods) and the nanoparticles were characterized by XRD, SEM, DLS, FT-IR, and VSM techniques. SEM images showed MNPs, MNPs-COOH, and MNPs-COOH-CUR nanoparticles have average sizes of 55, 71, and 77 nm respectively and VSM results show a small decrease in the saturation magnetization of the MNP-COOH compared to MNP. Curcumin loading was studied and the different amounts of loading obtainable by various solvents were investigated; resulting in loading as high as 56.149 wt% in Water/Ethanol (30% v/v) as a solvent. The conjugation of Curcumin to MNP-COOH was confirmed by FT-IR analysis. The hydrodynamic diameter of MNP using DLS test was 37.7 nm. In MTT test, MNP-COOH-CUR exhibited potent anticancer activity and results in MRI showed this Nano-system can be used as a contract agent. MNP-COOH-CUR are promising magnetic drug carriers which can be simultaneously used as a contrast agent in MRI, magnetically targeted drug delivery systems, and other biomedical applications. This approach could be extended through further research to preclinical and clinical use and may play a significant role in future cancer treatment and cancer imaging technologies. In conclusion, the results presented in this study suggest that MNP-COOH-CUR show promise to be further evaluated for diagnostic and treatment purposes. 

## References

[1] Corot C, Robert P, Idée J-M, Port M (2006). Recent advances in iron oxide nanocrystal technology for medical imaging. Adv. Drug Del. Rev.

[2] Pankhurst Q A, Connolly J, Jones S, Dobson J (2003). Applications of magnetic nanoparticles in biomedicine. J. Phys. D: Appl. Phys.

[3] Dobson J (2006). Magnetic nanoparticles for drug delivery. Drug Dev. Res.

[4] Karimi Z, Karimi L, Shokrollahi H (2013). Nano-magnetic particles used in biomedicine: Core and coating materials. Mater. Sci. Eng., C.

[5] Cheraghipour E, Javadpour S, Mehdizadeh A R (2012). Citrate capped superparamagnetic iron oxide nanoparticles used for hyperthermia therapy. J. Biomed. Sci. Eng.

[6] Mourino MR (1991). From Thales to Lauterbur, or from the lodestone to MR imaging: magnetism and medicine. Radiology.

[7] Dai Tran L, Hoang NMT, Mai TT, Tran HV, Nguyen NT, Tran TD, Do M H, Nguyen QT, Pham DG, Ha TP (2010). Nanosized magnetofluorescent Fe3O4–curcumin conjugate for multimodal monitoring and drug targeting. Colloid. Surf. A Physicochem. Eng. Asp.

[8] Felice B, Prabhakaran MP, Rodríguez AP, Ramakrishna S (2014). Drug delivery vehicles on a nano-engineering perspective. Mater. Sci. Eng. C.

[9] Dong X, Ding Y, Wu P, Wang C, Schäfer CG (2017). Preparation of MRI-visible gadolinium methacrylate nanoparticles with low cytotoxicity and high magnetic relaxivity. J. Mater. Sci.

[10] Carvalho A, Martins MBF, Corvo ML, Feio G (2014). Enhanced contrast efficiency in MRI by PEGylated magnetoliposomes loaded with PEGylated SPION: Effect of SPION coating and micro-environment. Mater. Sci. Eng. C.

[11] Shi Y (2006). Superparamagnetic nanoparticles for magnetic resonance imaging (MRI) diagnosis.

[12] Chomoucka J, Drbohlavova J, Huska D, Adam V, Kizek R, Hubalek J (2010). Magnetic nanoparticles and targeted drug delivering. Pharmacol. Res.

[13] Du C-X, Zhang T-B, Dong S-L, Han L, Liang X-J, Li L-H, Wei Y (2016). A magnetic gene delivery nanosystem based on cationic liposomes. J. Mater. Sci.

[14] Neuberger T, Schöpf B, Hofmann H, Hofmann M, Von Rechenberg B (2005). Superparamagnetic nanoparticles for biomedical applications: possibilities and limitations of a new drug delivery system. J. Magn. Magn. Mater.

[15] Torchilin VP (2000). Drug targeting. Eur. J. Pharm. Sci.

[16] Berry CC (2009). Progress in functionalization of magnetic nanoparticles for applications in biomedicine. J. Phys. D: Appl. Phys.

[17] Nigam S, Barick K, Bahadur D (2011). Development of citrate-stabilized Fe3O4 nanoparticles: conjugation and release of doxorubicin for therapeutic applications. J. Magn. Magn. Mater.

[18] Lattuada M, Hatton TA (2007). Functionalization of monodisperse magnetic nanoparticles. Langmuir.

[19] Laurent S, Forge D, Port M, Roch A, Robic C, Vander Elst L, Muller RN (2008). Magnetic iron oxide nanoparticles: synthesis, stabilization, vectorization, physicochemical characterizations, and biological applications. Chem. Rev.

[20] Wagner S, Schnorr J, Pilgrimm H, Hamm B, Taupitz M (2002). Monomer-coated very small superparamagnetic iron oxide particles as contrast medium for magnetic resonance imaging: preclinical in vivo characterization. Invest. Radiol.

[21] Lestari MLAD, Indrayanto G, Harry, GB (2014). Chapter Three - Curcumin. Profiles of Drug Substances, Excipients and Related Methodology.

[22] Aggarwal BB, Kumar A, Bharti AC (2003). Anticancer potential of curcumin: preclinical and clinical studies. Anticancer Res.

[23] Naksuriya O, Okonogi S, Schiffelers RM, Hennink WE (2014). Curcumin nanoformulations: A review of pharmaceutical properties and preclinical studies and clinical data related to cancer treatment. Biomaterials.

[24] Xue X, Yu J-L, Sun D-Q, Zou W, Kong F, Wu J, Liu H-p, Qu X-J, Wang R-M (2013). Curcumin as a multidrug resistance modulator — A quick review. Biomed. Prev. Nutri.

[25] Maheshwari RK, Singh AK, Gaddipati J, Srimal RC (2006). Multiple biological activities of curcumin: A short review. Life Sci.

[26] Nguyen TTT, Ghosh C, Hwang S-G, Dai Tran L, Park JS (2013). Characteristics of curcumin-loaded poly (lactic acid) nanofibers for wound healing. J. Mater. Sci.

[27] Dilnawaz F, Singh A, Sahoo SK (2012). Transferrin-conjugated curcumin-loaded superparamagnetic iron oxide nanoparticles induce augmented cellular uptake and apoptosis in K562 cells. Acta Biomater.

[28] Konwarh R, Saikia JP, Karak N, Konwar BK (2010). ‘Poly (ethylene glycol)-magnetic nanoparticles-curcumin’trio: Directed morphogenesis and synergistic free-radical scavenging. Colloid. Surf. B. Biointerf.

[29] Li F, Li X, Li B (2011). Preparation of magnetic polylactic acid microspheres and investigation of its releasing property for loading curcumin. J. Magn. Magn. Mater.

[30] Yallapu MM, Othman SF, Curtis ET, Gupta BK, Jaggi M, Chauhan SC (2011). Multi-functional magnetic nanoparticles for magnetic resonance imaging and cancer therapy. Biomaterials.

[31] Wang N, Guan Y, Yang L, Jia L, Wei X, Liu H, Guo C (2013). Magnetic nanoparticles (MNPs) covalently coated by PEO–PPO–PEO block copolymer for drug delivery. J. Colloid Interface Sci.

[32] Cheng KK, Wang YX, Chow AH, Baum L (2014). Amyloid Plaques Binding Curcumin Conjugated Magnetic Nanoparticles For Diagnosis In Alzheimer›s Disease Tg2576 Mice. Alzheimers Dement.

[33] Mancarella S, Greco V, Baldassarre F, Vergara D, Maffia M, Leporatti S (2015). Polymer‐Coated Magnetic Nanoparticles for Curcumin Delivery to Cancer Cells. Macromol. Biosci.

[34] Schwertmann U, Cornell RM (2008). Iron oxides in the laboratory.

[35] Mascolo MC, Pei Y, Ring TA (2013). Room Temperature Co-Precipitation Synthesis of Magnetite Nanoparticles in a Large pH Window with Different Bases. Materials.

[36] Chen G, Ma Y, Su P, Fang B (2012). Direct binding glucoamylase onto carboxyl-functioned magnetic nanoparticles. Biochem. Eng. J.

[37] McCarthy SA, Davies G-L, Gun›ko YK (2012). Preparation of multifunctional nanoparticles and their assemblies. Nat. Protoc.

[38] Sun Y, Duan L, Guo Z, DuanMu Y, Ma M, Xu L, Zhang Y, Gu N (2005). An improved way to prepare superparamagnetic magnetite-silica core-shell nanoparticles for possible biological application. J. Magn. Magn. Mater.

[39] Riss TL, Moravec RA, Niles AL, Duellman S, Benink HA, Worzella TJ, Minor L (2016). Assay Guidance Manual.

[40] Tran H V, Dai Tran L, Nguyen TN (2010). Preparation of chitosan/magnetite composite beads and their application for removal of Pb (II) and Ni (II) from aqueous solution. Mater. Sci. Eng. C.

[41] Waldron RD (1955). Infrared Spectra of Ferrites. Physical Review.

[42] Bini RA, Marques RFC, Santos FJ, Chaker JA, Jafelicci Jr M (2012). Synthesis and functionalization of magnetite nanoparticles with different amino-functional alkoxysilanes. J. Magn. Magn. Mater.

[43] Yang K, Peng H, Wen Y, Li N (2010). Re-examination of characteristic FTIR spectrum of secondary layer in bilayer oleic acid-coated Fe 3 O 4 nanoparticles. Appl. Surf. Sci.

[44] Bhandari R, Gupta P, Dziubla T, Hilt JZ (2016). Single step synthesis, characterization and applications of curcumin functionalized iron oxide magnetic nanoparticles. Mater. Sci. Eng., C.

[45] Răcuciu M, Creangă D, Airinei A (2006). Citric-acid-coated magnetite nanoparticles for biological applications. Eur. Phys. J. E.

[46] Ngo TH, Do HM, Tran VH, Nguyen XP (2010). Facile and solvent-free routes for the synthesis of size-controllable Fe3O4 nanoparticles. Adv. Nat. Sci. Nanosci. Nanotech.

[47] Safee NHA, Abdullah MP, Othman MR (2010). Carboxymethyl chitosan-Fe3O4 nanoparticles: synthesis and characterization. Malays. J. Ana. Sci.

[48] Mohapatra S, Mallick S, Maiti T, Ghosh S, Pramanik P (2007). Synthesis of highly stable folic acid conjugated magnetite nanoparticles for targeting cancer cells. Nanotechnology.

[49] Liang S, Yang XZ, Du XJ, Wang HX, Li H J, Liu WW, Yao YD, Zhu YH, Ma YC, Wang J (2015). Optimizing the Size of Micellar Nanoparticles for Efficient siRNA Delivery. Adv. Funct. Mater.

[50] Salem M, Xia Y, Allan A, Rohani S, Gillies E R (2015). Curcumin-loaded, folic acid-functionalized magnetite particles for targeted drug delivery. RSC Advances.

